# Optimization, and *in vitro* and *in vivo* evaluation of etomidate intravenous lipid emulsion

**DOI:** 10.1080/10717544.2021.1917729

**Published:** 2021-05-07

**Authors:** Dandan Geng, Yan Li, Chunyan Wang, Bo Ren, Heping Wang, Chensi Wu, Yirong Zhang, Linlin Zhao, Ligang Zhao

**Affiliations:** a School of Pharmacy, North China University of Science and Technology, Tangshan, China; b Department of Pharmacy, Tangshan Maternal and Child Health Hospital, Tangshan, China; c Tangshan Key Laboratory of Novel Preparations and Drug Release Technology, Tangshan, China

**Keywords:** Etomidate, intravenous lipid emulsion, response surface methodology, pharmacokinetics, tissue distribution

## Abstract

The aim of this investigation was to develop an etomidate intravenous lipid emulsion (ETM-ILE) and evaluate its properties *in vitro* and *in vivo*. Etomidate (ETM) is a hydrophobic drug, and organic solvents must be added to an etomidate injectable solution (ETM-SOL) to aid dissolution, that causes various adverse reactions on injection. Lipid emulsions are a novel drug formulation that can improve drug loading and reduce adverse reactions. ETM-ILE was prepared using high-pressure homogenization. Univariate experiments were performed to select key conditions and variables. The proportion of oil, egg lecithin, and poloxamer 188 (F68) served as variables for the optimization of the ETM-ILE formulation by central composite design response surface methodology. The optimized formulation had the following characteristics: particle size, 168.0 ± 0.3 nm; polydispersity index, 0.108 ± 0.028; zeta potential, −36.4 ± 0.2 mV; drug loading, 2.00 ± 0.01 mg/mL; encapsulation efficiency, 97.65% ± 0.16%; osmotic pressure, 292 ± 2 mOsmol/kg and pH value, 7.63 ± 0.07. Transmission electron microscopy images showed that the particles were spherical or spheroidal, with a diameter of approximately 200 nm. The stability study suggested that ETM-ILE could store at 4 ± 2 °C or 25 ± 2 °C for 12 months. Safety tests showed that ETM-ILE did not cause hemolysis or serious vascular irritation. The results of the pharmacokinetic study found that ETM-ILE was bioequivalent to ETM-SOL. However, a higher concentration of ETM was attained in the liver, spleen, and lungs after administration of ETM-ILE than after administration of ETM-SOL. This study found that ETM-ILE had great potential for clinical applications.

## Introduction

**Figure 1.**
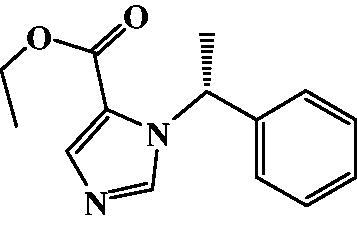
Structure of ETM.

Etomidate (ETM), (C_14_H_16_N_2_O_2_, Figure 1), is a non-barbiturate anesthetic agent, that acts on gamma aminobutyric acid-A receptors. It is also used as an anti-fungal agent, but has sedative and hypnotic effects in animals (Forman, 2011). ETM has little effect on the heart rate, stroke volume, and cardiac output; therefore, it is especially suitable for patients with cardiovascular diseases (Latson et al., 1992). In addition, ETM can reduce intracranial pressure, cerebral blood flow, and the cerebral metabolic rate of oxygen consumption in humans (Moss et al., 1979). Therefore, it has been used to maintain anesthesia during minor surgeries (Weng et al., 2013). The therapeutic index of ETM (26.0) is greater than that of thiopental sodium (4.6) and methohexital (9.5) (Janssen et al., 1975; Vanlersberghe & Camu, 2008). Moreover, the efficacy of ETM was found to be 12-fold higher than that of thiopental sodium, and it was found to be 1.8-fold more potent than propofol. Both thiopentone and propofol at equivalent doses have been shown to exhibit adverse cardiovascular effects (Gill & Scott, 1992). Therefore, ETM is generally used in specific clinical situations: rapid sequence induction in patients at risk of sepsis, procedural sedation, induction of general anesthesia for non-cardiac surgery, and induction of anesthesia in patients at cardiovascular risk (Erdoes et al., 2014). In a word, ETM is a very important anesthetic agent.

Currently, most formulations of ETM are injectable solutions and emulsions. In September 1982, an etomidate injectable solution (ETM-SOL) was first prepared by Hospira (Amidate^®^, prepared as a 20 mg/10 mL or 40 mg/20 mL solution). The formulation used 35% (v/v) propylene glycol as a co-solvent to dissolve ETM. However, transient venous pain was observed immediately after administration of ETM-SOL in approximately 20% of the patients (reported incidence was 1.2–42%) because of its strong acidity (pH = 6.0) and high osmotic pressure (OP) (Doenicke et al., 1999). Transient skeletal muscle movements were also noted in approximately 32% of the patients following ETM-SOL administration. A milky-white ETM oil-in-water injectable emulsion was developed (Braun, Melsungen, Germany) in May 2001, and marketed under the tradename Etomidate-Lipuro^®^ for injection (20 mg/10 mL). This did not contain propylene glycol so its OP was close to that of human plasma. Therefore, the incidence of adverse reactions caused by transient venous pain was considerably reduced. However, there have been a few reports on this product.

A lipid emulsion for intravenous administration was first successfully developed by Sweden in 1962 (Intralipid^®^). This did not contain a pharmaceutical drug, and was meant to serve as a source of calories and essential fatty acids for patients lacking adequate nourishment (Carpentier et al., 1997; Ton et al., 2005). With time, it was realized that an intravenous lipid emulsion could be used as a vehicle for hydrophobic drugs. Relative to other formulations, lipid emulsions have higher drug-loading capacities, improve drug stability, exhibit reduced toxicity and irritation, and are easy to manufacture. In addition, lipid emulsions use little or no organic solvent, so cause less irritation on administration than solutions (Li et al., 2013). However, an intravenous lipid emulsion is a thermodynamically unstable system (Wang et al., 2020), which can lead to stability issues such as increased droplet size, aggregation, flocculation, and demulsification. Therefore, it is necessary to design and optimize the formulation and evaluate its stability and safety.

The emulsifier is the most important component of an intravenous lipid emulsion. However, the stringent regulations permit the use of only a few types of emulsifiers in intravenous lipid emulsions. Soybean phospholipids and egg lecithin are widely used since they show good biocompatibility. In recent years, synthetic emulsifiers, such as poloxamer 188 (F68) and Solutol HS 15, have also been used in intravenous emulsions.

Although intravenous lipid emulsions—mainly used for the delivery of hydrophobic drugs—have been around for nearly 60 years, there are far fewer formulations of this type on the market than conventional dosage forms such as tablets and capsules. The present study aimed to optimize the formulation of ETM intravenous lipid emulsion (ETM-ILE) and evaluate its pharmacokinetic profile and tissue distribution in rats, using ETM-SOL as a reference.

## Materials and methods

### Materials and animals

ETM (> 99.5%) was purchased from Wuhan Dongkang Source Technology Co. Ltd. (Wuhan, China). Soybean phospholipid, egg lecithin (PL-100M^®^), and sodium oleate were purchased from A.V.T. Pharmaceutical Co. Ltd. (Shanghai, China). Soybean oil (SO), corn oil, and castor oil were purchased from Zhejiang Tianyushan Medicinal Oil Co. Ltd. (Zhejiang, China). Medium-chain triglycerides (MCT) and poloxamer 188 were obtained from Xi’an Tianzheng Medicinal Materials Co. Ltd. (Shaanxi, China). All chemicals and reagents were of analytical grade, except for methanol, which was of high performance liquid chromatography (HPLC) grade. Ultrapure water was produced in the laboratory.

Male Sprague-Dawley rats (200 ± 20 g) and male Japanese white rabbits (2.5 ± 0.5 kg) were obtained from Beijing HFK Bioscience Co. Ltd. (Beijing, China). The animal license numbers were 2019–0004 and 2019–0008, respectively. The animal experiments were approved by the Institutional Animal Ethics Committee of North China University of Science and Technology (Tangshan, China). All animals were treated humanely and suffering was alleviated.

### Solubility of ETM

Excess ETM powder was added to 5 mL of corn oil, castor oil, SO, MCT, SO-MCT mixture, and phosphate buffer solutions (PBS) of pH 3–10. The mixtures were vibrated for 36 h at 25 °C using the constant temperature gas bath oscillator. The supernatant was collected and filtered through a 0.45 μm membrane. The filtrate was subjected to HPLC to determine the concentration of ETM.

### Single factor experiments

Four kinds of oil (SO, MCT, refined corn oil, and castor oil) and three types of emulsifiers (soybean phospholipid, egg lecithin, and F68) were used to investigate the influence of these factors on the properties of ETM-ILE, including droplet size, polydispersity index (PDI), zeta potential (ZP), and drug loading (DL). Furthermore, the process of homogenization had a significant influence on particle size and PDI, so the effect of homogenization at 700, 1000, and 1300 bar was investigated.

### Design of experiment

Central composite design response surface methodology (CCD-RSM) was used to design the experiments for the optimization of the ETM-ILE formulation. Based on the results of the single-factor experiments, the independent variables were the proportion of oil (5–30%), egg lecithin (0.6–1.8%), and F68 (0.1–0.5%) used. The primary aim was to minimize the droplet size of ETM-ILE. In the range of 20–200 nm, the small droplet size can resist the physical destabilization caused by gravitational separation, flocculation and/or coalescence (Bernardi et al., 2011), which is the principle of formulation of nanoemulsions. Moreover, small particle sizes can also allow faster drug absorption and thus improve bioavailability (Wu et al., 2006). The CCD design consisted of 20 runs, as shown in Table 1.

**Table 1.** Experimental design by CCD-RSM.

Experiment number	Oil (%)	Egg lecithin (%)	F68 (%)	Droplet size (nm)
1	17.50	1.20	0.50	207.3
2	17.50	1.20	0.30	213.1
3	24.93	0.84	0.42	258.2
4	17.50	0.60	0.30	252.8
5	17.50	1.20	0.30	213.4
6	17.50	1.20	0.30	211.5
7	10.07	1.56	0.18	178.5
8	24.93	1.56	0.42	223.3
9	10.07	0.84	0.42	202.1
10	17.50	1.20	0.10	229.6
11	10.07	1.56	0.42	174.7
12	30.00	1.20	0.30	258.9
13	5.00	1.20	0.30	162.9
14	24.93	0.84	0.18	278.7
15	17.50	1.20	0.30	211.7
16	17.50	1.20	0.30	210.5
17	24.93	1.56	0.18	235.5
18	17.50	1.80	0.30	196.6
19	17.50	1.20	0.30	213.4
20	10.07	0.84	0.18	211.0

### Preparation of ETM-ILE

ETM-ILE was prepared by high-pressure homogenization. ETM and egg lecithin were dissolved in the oil phase, while in the aqueous phase, sodium oleate, glycerol and F68 were dissolved in water for injection. For both phases, solubilization was ultrasound-assisted, and carried out at 50 °C. Subsequently, the oil phase was slowly added to the aqueous phase, with stirring at 500 rpm for 10 min, using a MYP11-2A magnetic stirrer (IKA Company, Staufen, Germany). A coarse emulsion was prepared using a Fluko^®^ FA25 microfluidizer (Fluko Equipment Shanghai Co. Ltd, Shanghai, China) operated at 13,000 rpm for 10 min. Finally, ETM-ILE was prepared by M-110EH high-pressure homogenization (Microfluidics Corporation, Westwood, MA, USA). The emulsion was sealed in 10 mL penicillin bottles flushed with nitrogen, and sterilized by high-pressure steam at 121 °C for 15 min.

### Quality evaluation

#### Measurement of droplet size, PDI and ZP

Photon correlation spectroscopy (PCS, Zetasizer Nano ZS90 analyzer, Malvern Instruments Co., Worcestershire, UK) was used for the measurement of these three parameters. ETM-ILE was diluted 1:5000 (v/v) with purified water for the evaluation of droplet size, PDI, and ZP. The ZP of the emulsion is indicative of its stability: high surface charge results in stronger inter-particulate repulsion and less aggregation of particles (Elmowafy et al., 2020). Transmission electron microscopy (TEM, JEM-2800F, Tokyo, Japan) was used to observe the size and morphology of the droplets. ETM-ILE was diluted 1:100 000 (v/v) with purified water and dropped on a copper wire. The samples were observed at 120.0 kV after drying.

#### Measurement of DL and encapsulation efficiency (EE)

The concentration of ETM was analyzed by HPLC using a Shimadzu LC-20AT system. The mobile phase consisting of 40% water and 60% methanol was pumped through an Agilent C_18_ column (particle size 5 μm, 4.6 × 150 mm) at a flow rate of 1.0 mL/min. ETM was detected at a wavelength of 247 nm. Quantification was performed using propyl paraben as an internal standard. The column temperature was maintained at 40 °C, and the injection volume was 20 μL. ETM-ILE (100 μL) was diluted 100-fold with methanol to break the emulsion. Took 300 μL added into 300 μL of internal standard solution, mixed until uniform. Then the content of ETM was analyzed by HPLC. The DL was calculated based on the Equation (1):

(1) $$\mathrm{DL}=\frac{c}{0.1}\times 10\;$$

Here, *c* is the concentration analyzed by HPLC, the units of *c* and DL are both mg/mL.

ETM in the dispersed and continuous phases was isolated using a Sephadex G-50 gel column (10 × 100 mm). ETM-ILE (300 μL) or ETM-SOL (300 μL) was added to a Sephadex G-50 gel column and eluted with distilled water. Absorbance was measured at 247 nm for every 2 mL of eluent, and the elution volume of the dispersed phase was determined. The drugs in the dispersed phase were collected and analyzed by HPLC (Zhang et al., 2013). The EE (%) was calculated as follows:

(2) $$\mathrm{EE}(\%)\;=\;\frac{w_{d}}{w_{t}}\;\times \;100\;$$

Here, *w_d_* represents the weight of ETM in the dispersed phase and *w_t_* represents the total weight of ETM in the emulsion.

#### Measurement of OP and pH value

The OP of ETM-ILE was measured using a 3250 cryoscopic osmometer (Advanced Instruments, USA), calibrated with 100, 290 and 1500 mOsmol/kg standard solutions. ETM-ILE was kept at room temperature for 10 min, and transferred into the sample tube for OP measurement.

The pH meter (SevenCompact™ S220, METTLER TOLEDO, Switzerland) was used to measure the pH of ETM-ILE at room temperature. Each sample was determined three times.

### Stability study

The long-term stability test (4 ± 2 °C) and accelerated stability test (25 ± 2 °C) were investigated for a period of 12 months. The samples were determined the changes of parameters such as droplet size, PDI, ZP, DL, EE and pH respectively.

### Hemolysis experiment

Blood was collected from the ear marginal veins of rabbits and centrifuged at 2500 rpm for 10 min. The plasma was discarded and the cells were washed with normal saline until the supernatant was clear. A 2% (v/v) suspension of erythrocytes in normal saline was then prepared.

The erythrocyte suspension, 0.9% normal saline, distilled water, and ETM-ILE were filled into tubes labeled 1–7, respectively, as shown in Table 2. Tube number 1–5 were the samples to be testes. Tube number 6 was the negative control, and number 7 tube was the positive control. The samples were kept at 37 ± 0.5 °C for 3 h to observe hemolysis.

**Table 2.** Samples for *in vitro* evaluation of the effect of ETM-ILE on hemolysis.

Number	1	2	3	4	5	6	7
2% erythrocyte suspensions (mL)	2.5	2.5	2.5	2.5	2.5	2.5	2.5
0.9% normal saline (mL)	2.0	2.1	2.2	2.3	2.4	2.5	0
Distilled water (mL)	0	0	0	0	0	0	2.5
ETM-ILE (mL)	0.5	0.4	0.3	0.2	0.1	0	0
Results	–	–	–	–	–	–	+

– Indicates nonhemolytic and + indicates hemolytic.

### Vascular irritation study

The rabbits were randomly divided into two groups (*n* = 3). ETM-ILE or ETM-SOL (1.0 mg/kg) was injected into the right ear margin vein and the same volume of 0.9% normal saline was injected into the left ear margin vein. The rabbits were sacrificed 24 h after administration, and the ear tissues were cut off and fixed with 10% formaldehyde solution. Morphological changes in the blood vessels were observed by hematoxylin-eosin (HE) staining.

### Study of in vivo pharmacokinetics

The rats were fasted for 12 h with free access to water prior to the experiments (Ahmed et al., 2018). They were randomly divided into two groups (*n* = 6) and were intravenously administered either ETM-ILE or ETM-SOL (5.0 mg/kg) via the tail vein. Blood samples (0.5 mL) were collected from the jugular vein at 2, 5, 10, 15, 25, 35, 45, 60, 120, and 180 min. The samples were centrifuged at 10,000 rpm for 10 min to separate plasma. This was collected and stored at −80 °C.

### Tissue distribution study

The rats were randomly divided into two groups, with each group containing 36 animals (6 rats each for 6 time points). ETM-SOL or ETM-ILE was administered via the tail vein at a dose of 5.0 mg/kg. The rats were humanely sacrificed at 2, 5, 10, 30, 60, and 120 min after drug administration. The brain, heart, liver, spleen, lungs, and kidneys were collected immediately. Each tissue was washed with ice-cold saline, cut into pieces, weighed, and homogenized thrice with 0.9% normal saline (w/v) (Shi et al., 2010; Gou et al., 2017). The samples were centrifuged at 5,000 rpm for 15 min. Took 100 µL of the homogenized solution, 20 μL of internal standard solution and 400 μL methanol were added. The concentration of ETM was quantified by HPLC.

## Results and discussion

### Solubility of ETM

**Figure 2.**
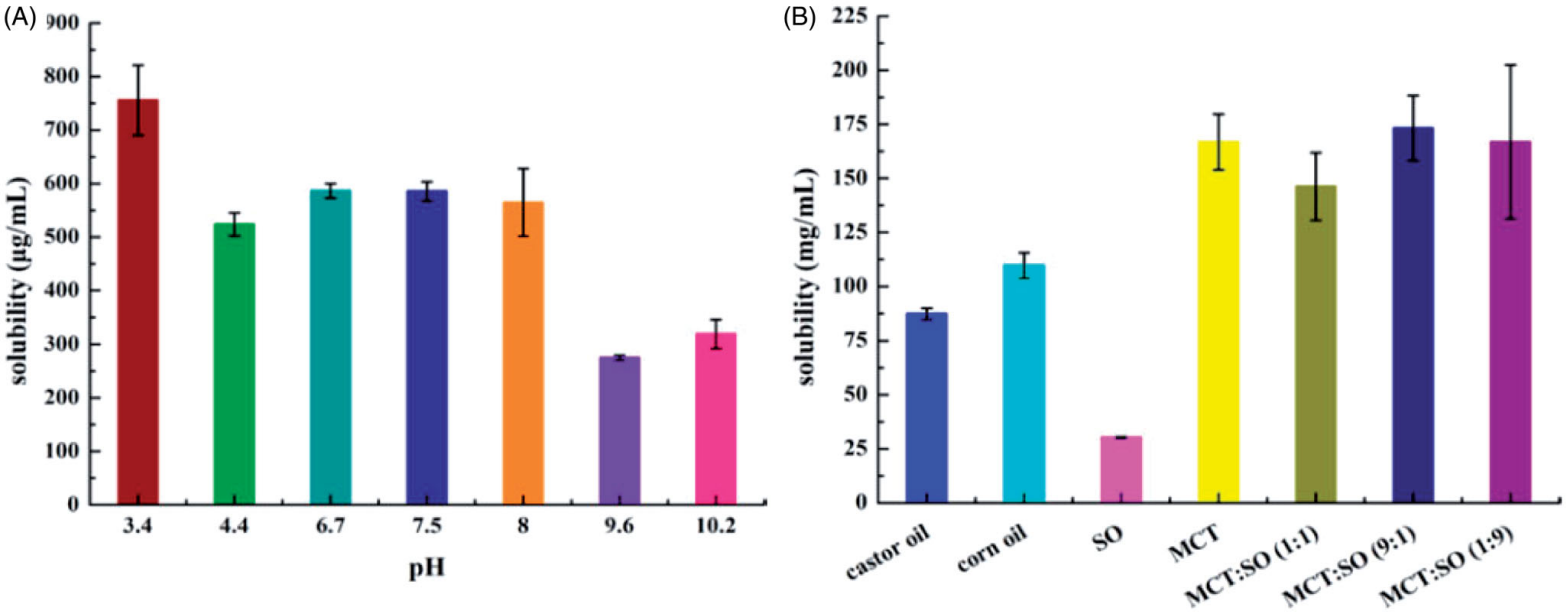
Solubility of ETM in PBS (A) and different oils (B) (*n* = 3).

Figure 2(A) summarizes the effect of the pH of PBS on ETM solubility. ETM has a high solubility in acidic solutions, which may be attributed to the weak basicity of the imidazole ring that can form salts in solution. However, the solubility of ETM in PBS did not meet the DL concentration (2.0 mg/mL). ETM exhibited lower solubility in alkaline solution. But it exhibited better solubility in the oils (Figure 2(B)) than in PBS, owing to its lipophilic nature. Dissolving ETM in an appropriate oil can help improve DL.

### Single factor experiments

#### Process of homogenization

**Figure 3.**
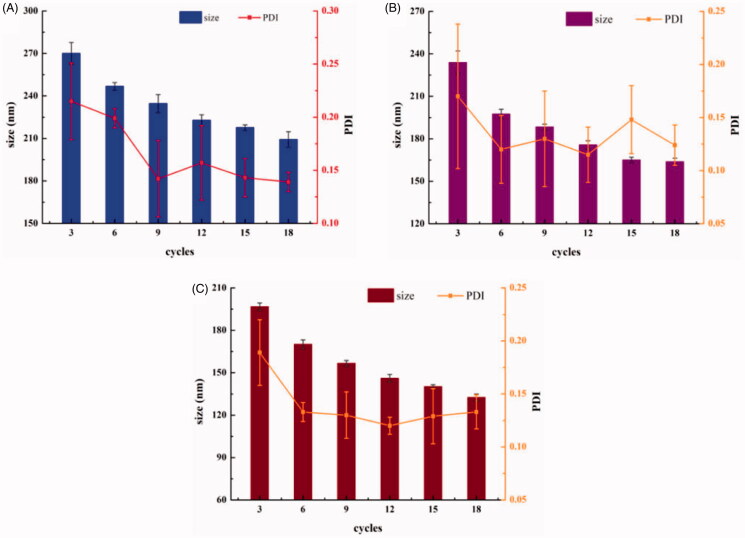
Effect of pressure and the number of cycles in high pressure homogenization on particle size and PDI (A: 700 bar; B: 1000 bar; C: 1300 bar) (*n* = 3).

High-pressure homogenization is an important step in the preparation of lipid emulsions. The pressure and number of cycles directly affected the particle size and PDI. The results are shown in Figure 3. A particle size of 209.2 nm was attained after 18 cycles at 700 bar, and 196.7 nm was attained after 3 cycles at 1300 bar. Taking into account the effect of abrasion and the ease operation of the machinery, we decided to run 6 cycles at a pressure of 1000 bar, to attain the target particle size of approximately 200 nm.

#### Choice of oil

**Figure 4.**
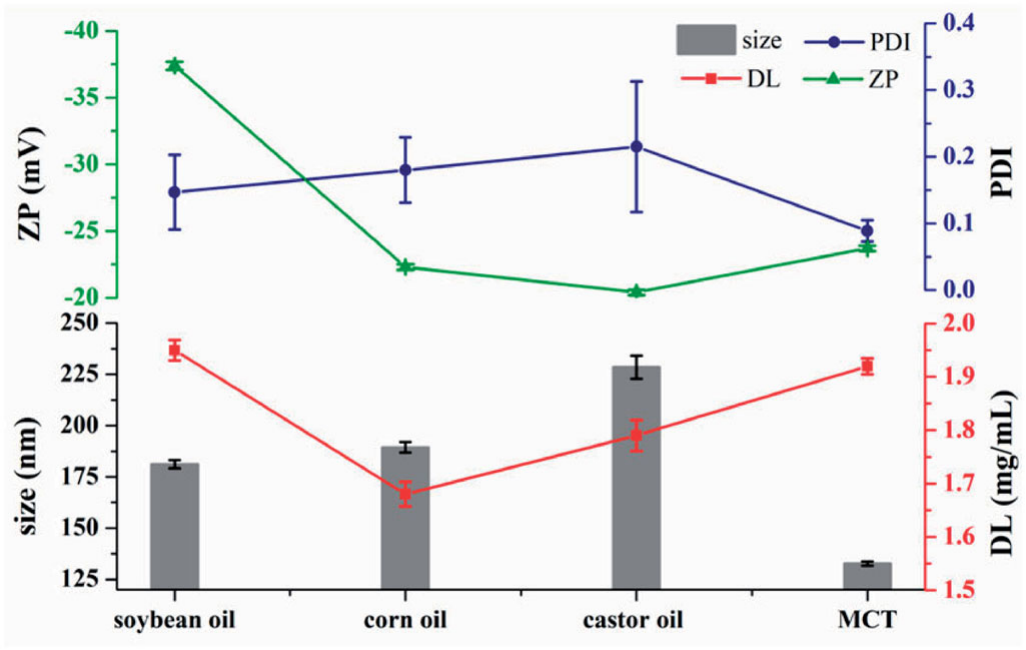
Properties of ETM-ILE prepared using different oil phases (*n* = 3).

SO, corn oil, castor oil, and MCT were used as the oil phases. The particle size, PDI, ZP, and DL of ETM-ILE were measured. The results are shown in Figure 4. The ETM-ILE prepared using castor oil had the highest viscosity and the largest particle size, therefore, castor oil was not suitable for intravenous injection. SO and corn oil are long-chain triglycerides. SO has been widely used in lipid emulsions, and its surface-active impurities contribute to the stability of emulsions (Powell et al., 2017). MCT is composed of shorter carbon chains, and the ETM-ILE prepared using MCT exhibited good fluidity, small particle size and PDI.

The solubility data shows that all of these oils could meet the DL requirements of 2.0 mg/mL. S_SO_, S_MCT_, S_(MCT:SO 1:9)_, S_(MCT:SO 1:1)_, and S_(MCT:SO 9:1)_ were found to be 30.26 ± 0.32 mg/mL, 166.83 ± 12.86 mg/mL, 166.86 ± 35.51 mg/mL, 146.22 ± 15.67 mg/mL, and 173.21 ± 15.13 mg/mL, respectively, confirming previous reports that MCT could improve DL (Gou et al., 2016). In addition, MCT could modulate the stability of lipid membranes and their resistance to peroxidation (Anez-Bustillos et al., 2016; Luo et al., 2019). ETM-ILE prepared using a mixture of soybean oil or MCT had the high DL and ZP (Figure 4). However, MCT used alone may cause metabolic acidosis and adverse reactions in the nervous system (Raman et al., 2017). Therefore, a mixture of oils (MCT:SO = 1:1, w/w) was selected as the oil phase for this emulsion.

#### Choice of emulsifiers

Soybean phospholipid, egg lecithin, and F68 were evaluated as potential emulsifiers for this formulation. Both soybean phospholipids and egg lecithin are derived from natural sources. There was little difference in the appearance, particle size, and PDI of the emulsions prepared using these two emulsifiers, but the ETM-ILE prepared using egg lecithin exhibited a higher ZP. Emulsification using F68 was very slow, but it could stabilize the phase-interfaces in the emulsion (Powell et al., 2017). In addition, F68 had an effect on the treatment of vascular occlusion caused by blood cell aggregation, and could also promote drug transport across the blood-brain barrier, thus increasing drug bioavailability in the brain (Agrawal et al., 2015). Therefore, egg lecithin and F68 were selected as the emulsifiers for use in this study.

### CCD-RSM

Modeling and statistical analyses were performed using Design-Expert 6.0.11 (Stat-Ease, Inc., Minneapolis, USA). Variance analysis results showed that the quadratic model adequately expressed the relationships between the variables (*p* < .0001) (Table 3). There was only a 0.01% chance that a ‘Model *F*-Value’ could occur due to noise. The response variable, droplet size (Y), could be predicted using the quadratic polynomial model expressed in Equation (3). Variables X_1_, X_2_, and X_3_ represent the oil, egg lecithin, and F68, respectively.

(3) $$Y=268.951+5.783X_{1}-134.940X_{2}-158.548X_{3}-0.002X_{1}^{2}+37.662X_{2}^{2}+183.704X_{3}^{2}-0.858X_{1}X_{2}-2.828X_{1}X_{3}+39.480X_{2}X_{3}\;$$

**Table 3.** Analysis of variance (ANOVA) for the quadratic polynomial model.

Source	Sum of squares	Degree of freedom	Mean square	*F*-value	*p*-Value
Model	16165.96	9	1796.22	749.40	<.0001
X_1_-oil	11,185.99	1	11,185.99	4666.93	<.0001
X_2_-lecithin	3958.75	1	3958.75	1651.64	<.0001
X_3_-F68	503.27	1	503.27	209.97	<.0001
X_1_X_2_	41.40	1	41.40	17.27	.0020
X_1_X_3_	50.00	1	50.00	20.86	.0010
X_2_X_3_	22.45	1	22.45	9.36	.0120
X_1_^2^	0.11	1	0.11	0.044	.8381
X_2_^2^	331.14	1	331.14	138.16	<.0001
X_3_^2^	96.21	1	96.21	40.14	<.0001
Residual	23.97	10	2.40	–	–
Lack of fit	16.68	5	3.34	2.29	0.1926 (not significant)
Pure error	7.29	5	1.46	–	–
Cor. Total	16,189.93	19	–	–	–

In this case, X_1_, X_2_, X_3_, X_1_X_2_, X_1_X_3_, X_2_X_3_, X_2_^2^, and X_3_^2^ were significant model terms (*p* < .0001). The ‘Lack of Fit *F*-value’ of 2.29 implied that it was not significant relative to the pure error. The correlation coefficient *R^2^* was 0.9985, implying that only 0.15% of the variations were not justified by the fitted model. The adjust *R*^2^ value was 0.9972, which was close to *R*^2^, implying that only 0.28% of the total variation was not explained by the model. This confirms the suitability of the polynomial regression model.

**Figure 5.**
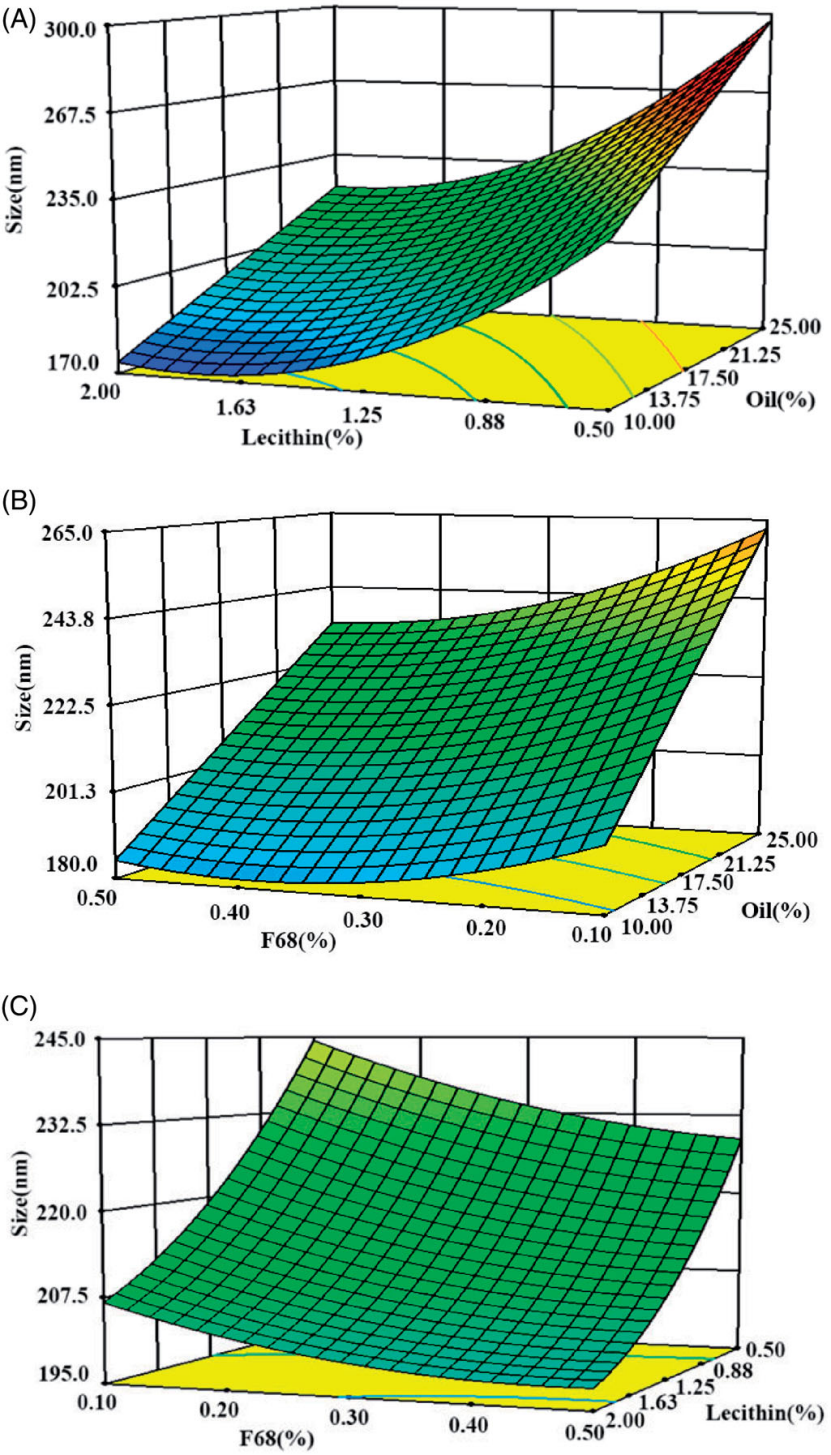
The CCD-RSM of three factors on the droplet size in ETM-ILE. (A: soybean oil and F68; B: egg lecithin and F68; C: egg lecithin and soybean oil).

The 3D plots depicted in Figure 5 show the way in which the particle size was affected by two variables when the third parameter was considered to be constant at the central point defined by the software.

The optimum formulation predicted by the software was as follows: ETM (0.2%, w/v), oil (10.07%, w/v, mixture of soybean oil and MCT, 1:1), egg lecithin (1.56%, w/v), F68 (0.34%, w/v), sodium oleate (0.05%, w/v), and glycerol (2.25%, w/v).

### Evaluation of ETM-ILE quality

The predicted and observed particle sizes were 173.2 nm and 168.0 nm, respectively, for the ETM-ILE prepared according to the optimized formulation. A bias of 3.10%, was calculated according to Equation (4).

(4) $$\mathrm{Bias}(\%)\;=\;\frac{\mathrm{observed}\;\mathrm{value}\;-\;\mathrm{predicted}\;\mathrm{value}}{\mathrm{observed}\;\mathrm{value}}\;\times \;100\;$$

**Figure 6.**
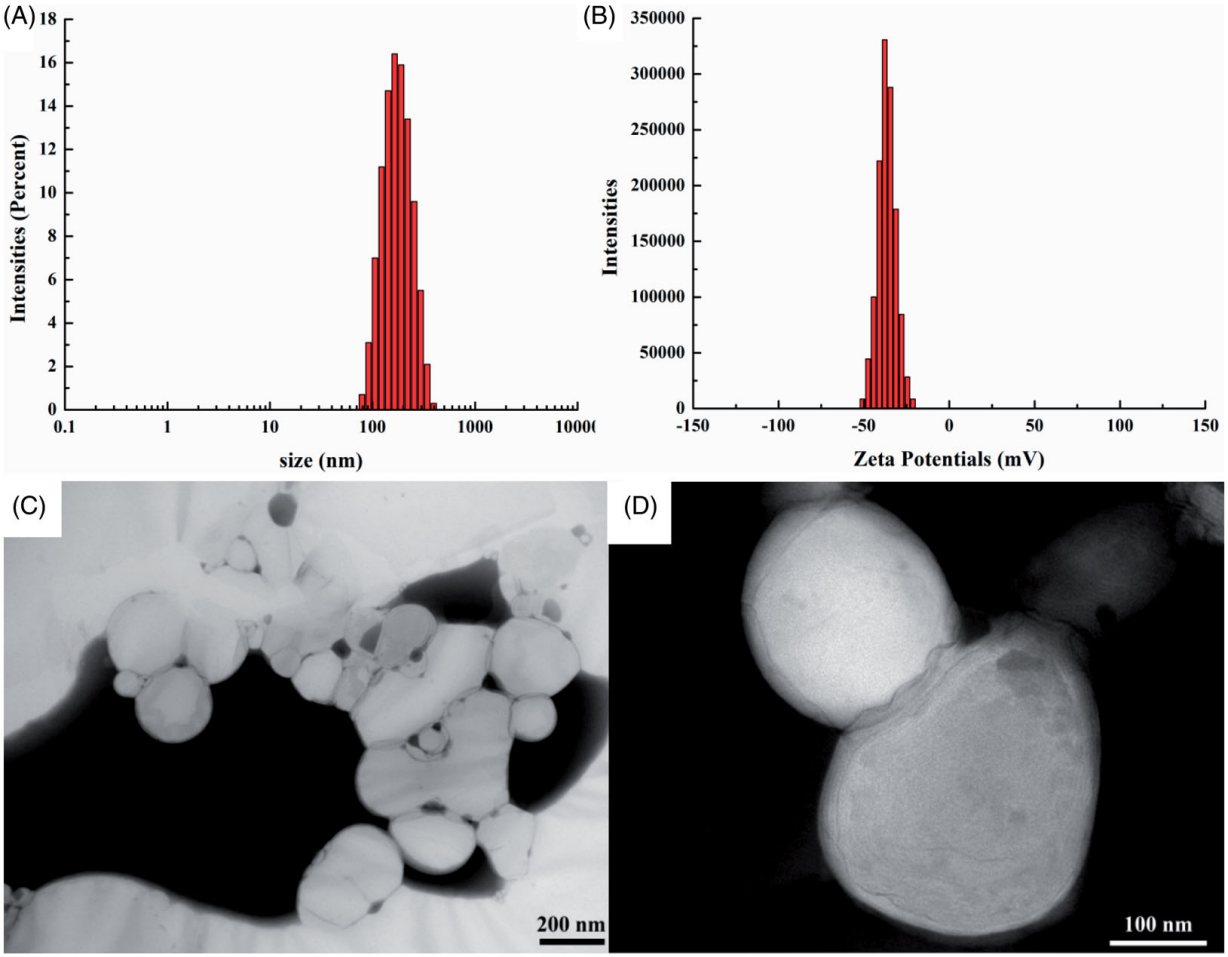
Characterization of ETM-ILE. (A) size detected by Zetasizer; (B) ZP detected by Zetasizer; (C) TEM image (scale bar 200 nm); (D) TEM image (scale bar 100 nm).

The particle size, PDI, ZP, DL, EE, OP and pH were found to be 168.0 ± 0.3 nm, 0.108 ± 0.028, −36.4 ± 0.2 mV, 2.00 ± 0.01 mg/mL, 97.65% ± 0.16%, 292 ± 2 mOsmol/kg, and 7.63 ± 0.07 respectively. The particle size and ZP of ETM-ILE, measured by the Zetasizer are shown in Figure 6(A–B). The TEM images are shown in Figure 6(C–D). The droplets had a smooth spherical appearance. The diameters of the droplets measured by TEM were consistent with the results described above.

### Stability study

The results of long-term stability test at 4 ± 2 °C and accelerated stability test at 25 ± 2 °C were shown in Table 4. The independent-samples *t*-test was used to analyze the results. No significant differences were found between the measured value and initial value at different time and temperature (*p* > 0.05). The appearance of ETM-ILE was the uniform milky white liquid without obvious oil-water separation and flocculation. The change of each parameter was within the acceptable range. Therefore, ETM-ILE was stable at 4 ± 2 °C or 25 ± 2 °C for at least 12 months.

**Table 4.** Stability study of ETM-ILE (mean ± SD, *n* = 3).

	Initial	Long-term stability test			Accelerated stability test		
		3 m	6 m	12 m	3 m	6 m	12 m
Size (nm)	168.0 ± 0.3	166.9 ± 1.7	170.2 ± 2.2	170.8 ± 2.6	168.9 ± 2.8	168.0 ± 1.5	170.4 ± 2.7
PDI	0.108 ± 0.028	0.112 ± 0.004	0.105 ± 0.009	0.109 ± 0.021	0.100 ± 0.014	0.111 ± 0.013	0.115 ± 0.012
pH	7.63 ± 0.07	7.56 ± 0.05	7.61 ± 0.02	7.60 ± 0.03	7.59 ± 0.04	7.57 ± 0.03	7.52 ± 0.04
ZP (mV)	−36.4 ± 0.2	−36.9 ± 0.3	−36.5 ± 0.5	−37.2 ± 0.9	−35.9 ± 0.8	−36.7 ± 0.4	−36.2 ± 0.6
DL (mg/mL)	2.00 ± 0.01	2.00 ± 0.02	2.00 ± 0.03	2.00 ± 0.04	2.00 ± 0.04	2.00 ± 0.04	1.97 ± 0.07
EE (%)	97.65 ± 0.16	97.66 ± 0.46	97.50 ± 0.35	97.33 ± 0.76	97.43 ± 0.26	97.53 ± 0.43	97.17 ± 0.35

### Hemolysis experiment

**Figure 7.**
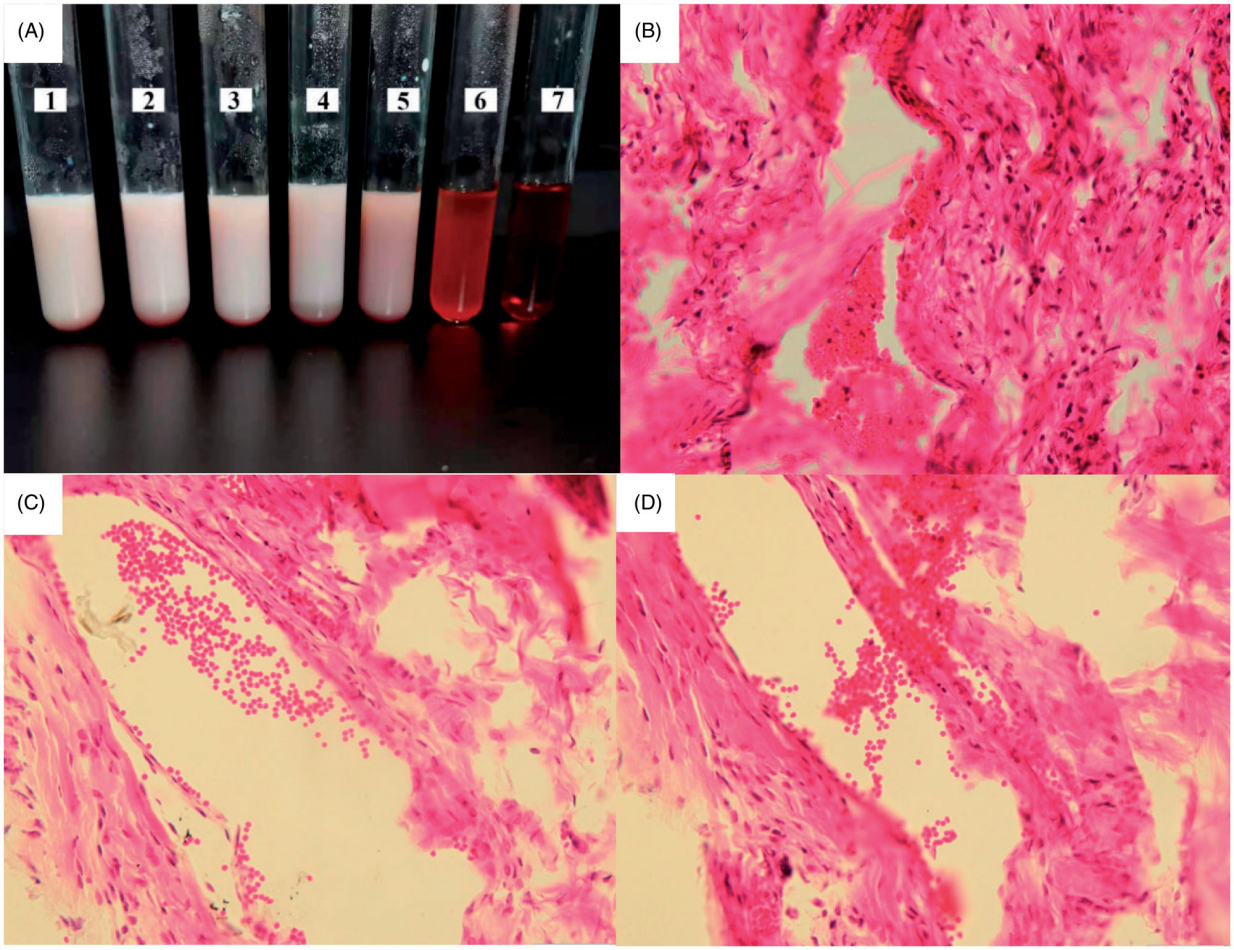
Safety tests for ETM-ILE: (A) *In vitro* hemolysis test for ETM-ILE, tubes 1–5 were experimental group, tube 6 was the negative control, and tube 7 was the positive control; Images of the pathological sections of rabbit ear at the site of administration (HE staining, 200× magnification) of the three groups: ETM-SOL group (B), ETM-ILE group (C), and Normal saline group (D).

Red blood cells were deposited at the bottom of test tubes number 1 to 5, that results were consistent with that of negative control tube 6. While in tube 7, there was no red blood cells at the bottom (Figure 7(A)), indicating that ETM-ILE did not undergo hemolysis, therefore it could be used in animal experiments. ETM-SOL had a high osmolality (4965 mOsmol/kg), which could cause hemolysis on injection (Doenicke et al., 1997). In contrast, the OP of ETM-ILE was similar to that of plasma, which prevented the rupture of red blood cells.

### Vascular irritation study

After administration of ETM-SOL, ETM-ILE, and 0.9% normal saline, no obvious redness or swelling were observed at the site of injection in the groups. Inflammatory stimulation observed after HE staining is shown in Figure 7(B–D). The group administered ETM-SOL (Figure 7(B)) exhibited loose tissue, edema of the vascular wall, and a large influx of inflammatory cells, indicating that ETM-SOL was an irritant to the blood vessels. The group administered ETM-ILE (Figure 7(C)) exhibited slight vasodilation, similar to the normal saline group (Figure 7(D)), suggesting that ETM-ILE had a slight irritant effect on the blood vessels.

### In vivo pharmacokinetic profile

**Figure 8.**
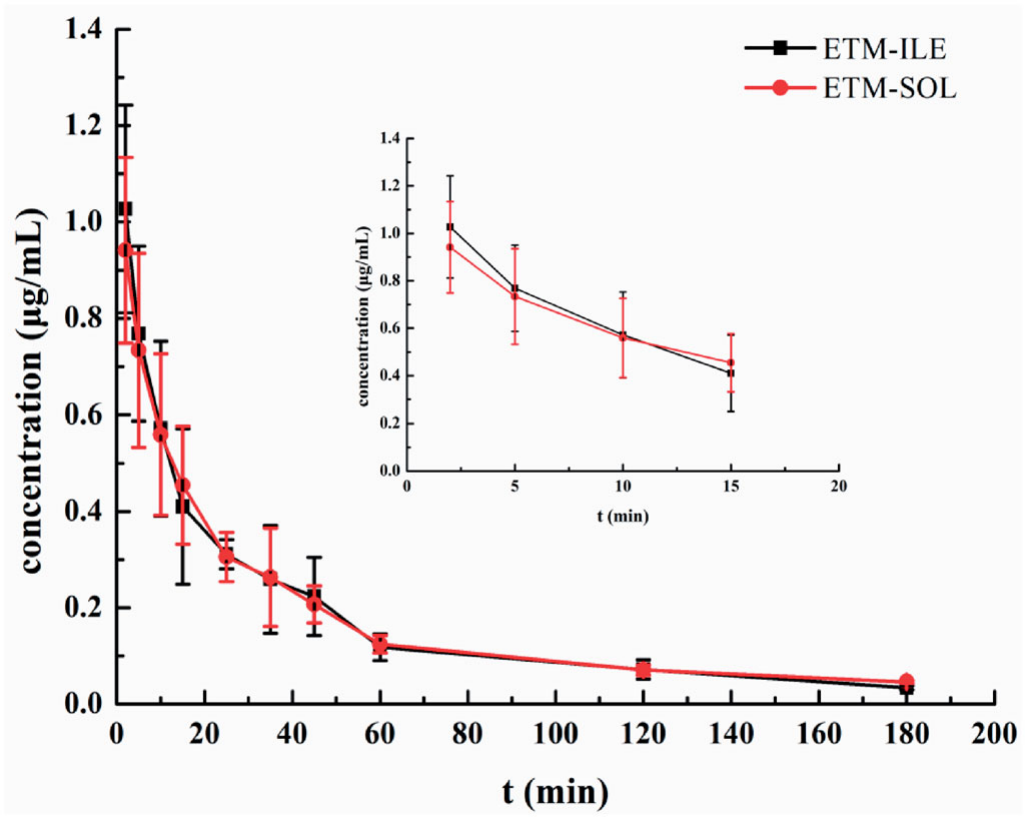
Mean plasma concentration-time profile of ETM after administration of ETM-ILE and ETM-SOL at a dose of 5.0 mg/kg (*n* = 6).

The mean plasma concentration-time profiles of ETM-ILE and ETM-SOL are shown in Figure 8. ETM in both formulations was rapidly eliminated from 2 min after administration, and could not be detected after 180 min. The pharmacokinetic parameters determined by the non-compartment model using the WinNonlin software package (Pharsight) are presented in Table 5. There were no significant differences in pharmacokinetic parameters for the two formulations (*p* > .05). The results indicated pharmacokinetic bioequivalence between ETM-ILE and ETM-SOL. It has been reported that lipophilic drugs are released rapidly from lipid emulsions, since it is a diffusion-controlled process, and the barrier effect of the oil-water interface is negligible (Salmela & Washington, 2014). The plasma protein binding rate of ETM was found to be 76%, which may also promote drug release from the lipid emulsion into plasma (Meuldermans & Heykants, 1976). ETM-ILE enters the mononuclear phagocyte system or is metabolized as endogenous chylomicrons, and cleared from the blood owing to its small particle size. The incorporation of MCT in ETM-ILE resulted in faster clearance (Dong et al., 2013). These factors may explain the similar pharmacokinetic profiles of ETM-ILE and ETM-SOL in rats.

**Table 5.** Pharmacokinetic parameters of ETM-ILE and ETM-SOL after administration at a dose of 5.0 mg/kg (mean ± SD, *n* = 6).

Parameters	ETM-ILE	ETM-SOL
*T*_max_ (h)	0.04 ± 0.02	0.05 ± 0.03
*C*_max_ (μg/mL)	1.05 ± 0.24	0.98 ± 0.17
*t*_1/2_ (h)	0.94 ± 0.07	1.14 ± 0.30
AUC_(0-t)_ (h·μg/mL)	0.52 ± 0.07	0.51 ± 0.07
MRT_(0-t)_ (h)	0.73 ± 0.09	0.78 ± 0.05
Cl (L/h/kg)	8.92 ± 1.19	8.45 ± 0.93

### Tissue distribution

**Figure 9.**
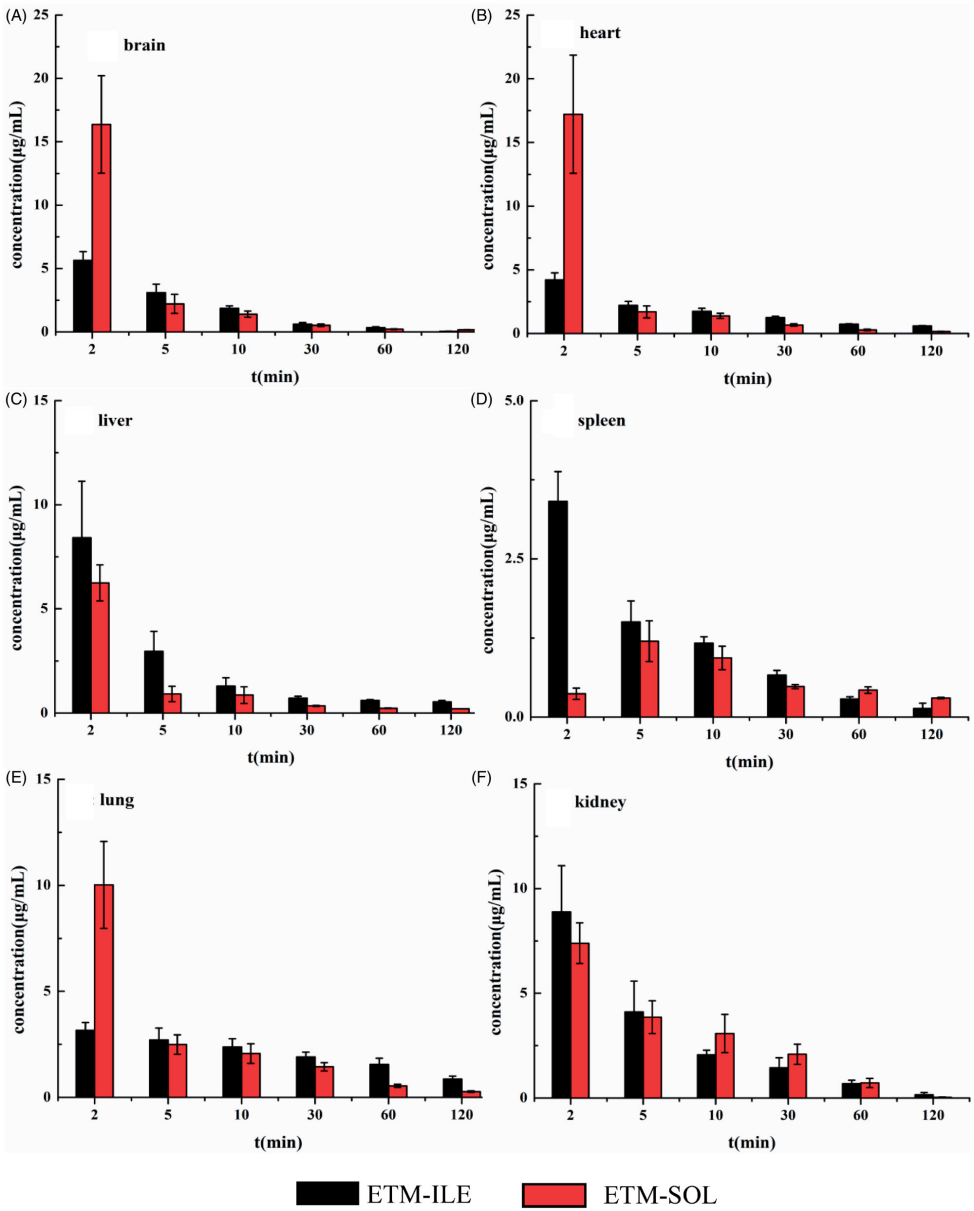
Tissue distribution of ETM after administration of ETM-ILE and ETM-SOL at doses of 5.0 mg/kg (*n* = 6).

The mean concentration-time profiles of ETM in different tissues after intravenous administration of ETM-ILE and ETM-SOL are shown in Figure 9, and the area under the curve (AUC_0−∞_) values in various tissues are shown in Table 6.

**Table 6.** Comparison of tissue AUCs of ETM after administration of ETM-ILE and ETM-SOL at a dose of 5.0 mg/kg (mean ± SD, *n* = 6).

	AUC_(0-∞)_ (h·μg/mL)	
Tissue	ETM-ILE	ETM-SOL
Brain	1.52 ± 0.11*	2.99 ± 1.10
Heart	3.11 ± 0.10	3.37 ± 1.33
Liver	4.48 ± 1.60*	1.75 ± 0.40
Spleen	1.86 ± 0.25*	1.18 ± 0.20
Lung	4.90 ± 0.71*	2.83 ± 0.35
Kidney	2.68 ± 0.30	2.85 ± 0.17

*Indicates AUC_(0-∞)_ of ETM-ILE is significantly different from ETM-SOL, *p* < 0.05.

Lipid emulsions changed the distribution of ETM in rats: at 2 min post-injection of ETM-ILE, high concentrations of ETM were observed in six different tissues, with the highest concentration being found in the kidneys, followed by the liver, brain, heart, spleen, and lungs. Meanwhile, following administration of ETM-SOL, the highest drug concentration after 2 min was found in the heart, followed by the brain, lungs, kidneys, liver, and spleen. Compared with ETM-SOL, the drug concentration in the brain, heart, and lung decreased after 2 min of administration of ETM-ILE, but the concentration in the liver, spleen, and kidneys increased. Unlike other organs, the maximum concentration was detected in spleen at 2 min after administration of ETM-ILE and 5 minutes after administration of ETM-SOL. This could be attributed to the droplet size and composition of ETM-ILE. Unmodified nanoparticles are usually taken up by the liver, spleen, and other parts of the reticuloendothelial system (RES) (Moghimi & Szebeni, 2003). Contrarily, the negative surface charge of ETM-ILE droplets could prevent clearance by the RES (Xiao et al., 2011). The AUC_0-∞_ for ETM-ILE was 2.56, 1.58, and 1.73-fold better in the liver, spleen, and lungs, respectively, compared to ETM-SOL. Interestingly, the AUC_0-∞_ of ETM-SOL in the brain was higher than that of ETM-ILE at 2 minutes point after administration. It is reasonable to assume that ETM is a small molecule with high lipophilic characteristics, which makes it can penetrate cross the blood-brain barrier (BBB) quickly. However, the concentration of ETM-ILE in the brain depended on the penetration speed of oil droplets with large volume in the lipid emulsion. It is reported that the BBB can prevent many water-soluble molecules and even lipid-soluble molecules with large molecular weight from entering the brain freely (Lin et al., 2012). Additionally, there is a repulsive force between the charge on the surface of the particles and the charge on brain endothelial cells, which also could prevent ETM in oil droplets from crossing the BBB (Shi et al., 2010). The FDA has warned that the repeated use of general anesthetic drugs may affect brain development in children (Ganzberg, 2017). As an anesthetic drug, excessive ETM entering the brain is unfavorable for patients with cardiovascular and cerebrovascular diseases. In animal experiments, it was observed that it took a long time for rats to regain consciousness after the administration of ETM-SOL, and there were symptoms of hematochezia.

## Conclusion

In this study, the optimal formula for ETM-ILE consisted of ETM (0.2%, w/v), oil (10.07%, w/v, mixture of soybean oil and MCT 1:1), egg lecithin (1.56%, w/v), F68 (0.34%, w/v), sodium oleate (0.05%, w/v) and glycerol (2.25%, w/v). The emulsion exhibited good physical and chemical properties. It could be stored stably for 12 months at 4 ± 2 °C or 25 ± 2 °C. Hemolysis and vascular irritation tests confirmed that ETM-ILE was safe for injection. The pharmacokinetic and tissue distribution studies were performed in rats, and the results showed that ETM-ILE exhibited a similar pharmacokinetic profile to ETM-SOL, but allowed greater drug distribution to the liver, spleen, and lungs. Consequently, this study suggests that ETM-ILE has great potential for clinical applications.

## Abbreviations

ANOVA: analysis of variance
AUC: area under the concentration time profile
CCD-RSM: central composite design-response surface methodology
*C*_max_: the peak concentration
Cl: clearance
DL: drug loading
ETM: etomidate
ETM-ILE: etomidate intravenous lipid emulsion
ETM-SOL: etomidate injectable solution
EE: encapsulation efficiency
F68: poloxamer 188
HE: hematoxylin-eosin
HPLC: high performance liquid chromatography
MCT: medium chain triglycerides
MPS: mononuclear phagocyte systems
MRT: mean residence time
OP: osmotic pressure
PBS: phosphate buffer solutions
PCS: photon correlation spectroscopy
PDI: polydispersity index
SO: soybean oil
TEM: transmission electron microscopy
*T*_max_: the peak time
*t*_1/2_: half time
ZP: zeta potential
